# Supplementary material for: Novel Driver Strength Index highlights important cancer genes in TCGA PanCanAtlas patients
Source: PeerJ. 2022 Aug 11;10:e13860. doi: 10.7717/peerj.13860 (PMC9375969; doi:10.7717/peerj.13860)
Supplement: Supplemental Information 2 [file peerj-10-13860-s002.zip › COHORTS/genes plots/cohorts/2021_8_16_14_9_distribution_cohorts_GBM.pdf]

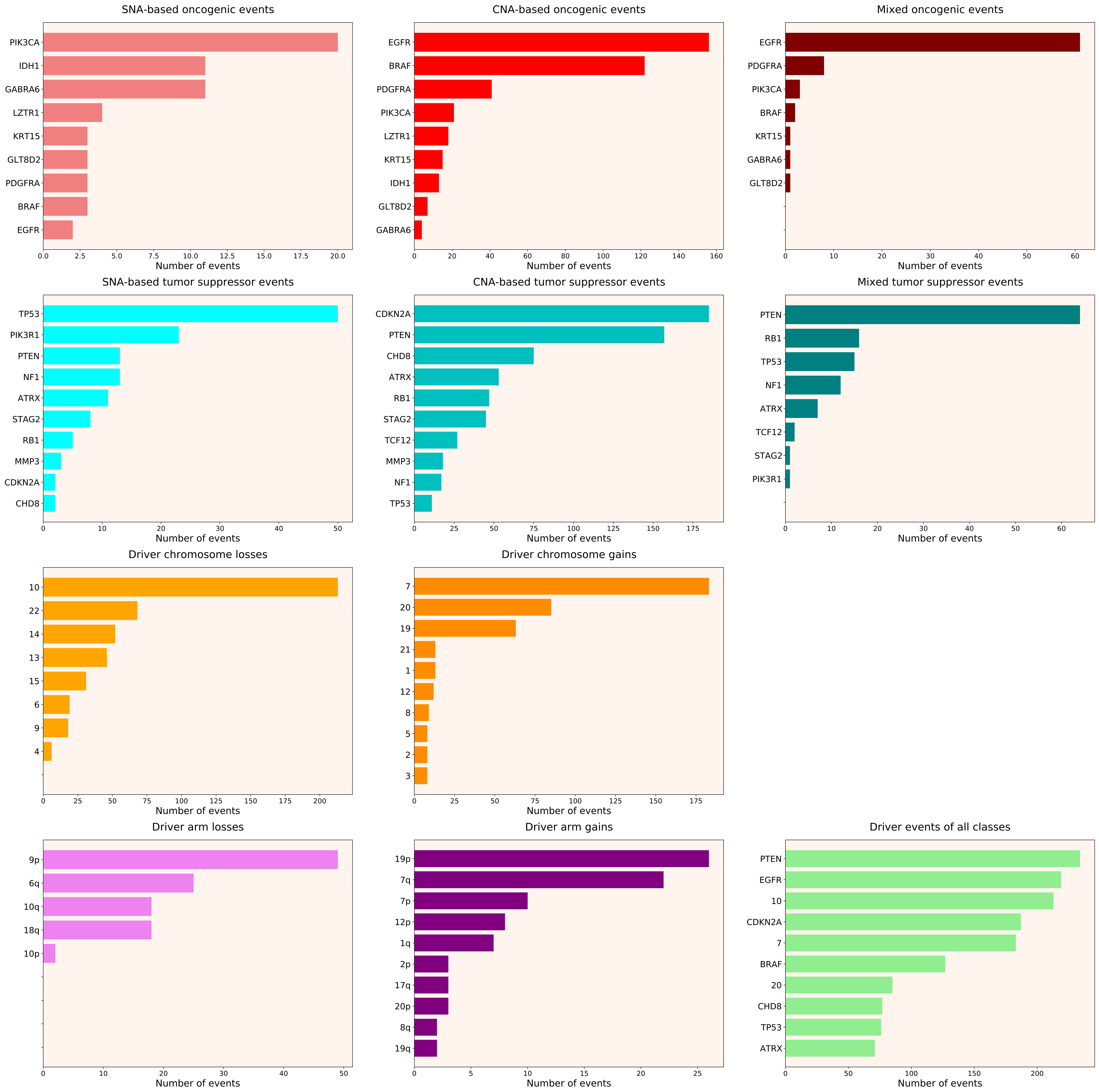
SNA-based oncogenic events
PIK3CA
IDH1
GABRA6
LZTR1
KRT15
GLT8D2
PDGFRA
BRAF
EGFR
Number of events
CNA-based oncogenic events
EGFR
BRAF
PDGFRA
PIK3CA
LZTR1
KRT15
IDH1
GLT8D2
GABRA6
Number of events
Mixed oncogenic events
EGFR
PDGFRA
PIK3CA
BRAF
KRT15
GABRA6
GLT8D2
Number of events
SNA-based tumor suppressor events
TP53
PIK3R1
PTEN
NF1
ATRX
STAG2
RB1
MMP3
CDKN2A
CHD8
Number of events
CNA-based tumor suppressor events
CDKN2A
PTEN
CHD8
ATRX
RB1
STAG2
TCF12
MMP3
NF1
TP53
Number of events
Mixed tumor suppressor events
PTEN
RB1
TP53
NF1
ATRX
TCF12
STAG2
PIK3R1
Number of events
Driver chromosome losses
10
22
14
13
15
6
9
4
Number of events
Driver chromosome gains
7
20
19
21
1
12
8
5
2
3
Number of events
Driver arm losses
9p
6q
10q
18q
10p
Number of events
Driver arm gains
19p
7q
7p
12p
1q
2p
17q
20p
8q
19q
Number of events
Driver events of all classes
PTEN
EGFR
10
CDKN2A
7
BRAF
20
CHD8
TP53
ATRX
Number of events
